# Cardiometabolic risk factors and disease trends for atrial fibrillation in individuals with type 1 diabetes: a nationwide registry study

**DOI:** 10.1186/s12933-024-02561-z

**Published:** 2025-03-12

**Authors:** Lara El Khalili, Linn El Khalili, Araz Rawshani, Jan Borén, Deepak L. Bhatt, Hertzel C. Gerstein, Darren K. McGuire, Edvin Helleryd, Elmir Omerovic, Björn Eliasson, Truuls Råmunddal, Naveed Sattar, Aidin Rawshani

**Affiliations:** 1 Department of Molecular and Clinical Medicine, Institute of Medicine, University of Gothenburg, Gothenburg, Sweden; 2 Wallenberg Laboratory for Cardiovascular and Metabolic Research, Institute of Medicine, University of Gothenburg, Gothenburg, Sweden; 3 Icahn School of Medicine at Mount Sinai Health System, New York, USA; 4 Population Health Research Institute, McMaster University and Hamilton Health Sciences, Hamilton, ON Canada; 5 Department of Internal Medicine, University of Texas Southwestern Medical Center, Dallas, TX USA; 6 Parkland Health and Hospital System, Dallas, TX USA; 7 Institute of Cardiovascular and Medical Sciences, British Heart Foundation Glasgow Cardiovascular Research Centre, London, UK; 8 Wallenberglaboratoriet, Sahlgrenska Universitetssjukhuset, Bruna Stråket 16, 413 45 Göteborg, Sweden

**Keywords:** Atrial fibrillation, Cardiometabolic disease, Type 1 diabetes mellitus, Cardiometabolic risk factors, Cardiovascular medicine, Population health

## Abstract

**Objective:**

To investigate standardized incidence of atrial fibrillation (AF) in individuals with type 1 diabetes (T1DMM) compared with matched controls from the general population. Additionally, to examine optimal levels- and relative importance of risk factors associated with AF and numbers of risk factors necessary to reduce excess risk in individuals with T1DM.

**Research design and methods:**

The study included individuals with T1DM between 2001 and 2019 and matched controls without T1DM. The outcome of interest was the first occurrence of AF. Standardized incidence rates and Cox regression were used for analyzing incidence and risk associations.

**Results:**

The study comprises analyses of data from 36,069 persons with T1DM and 165,705 matched controls; average age 34.1; 43.2% women. Incidence rates per 100,000 person years for AF in persons with T1DM declined between 2001 and 2019 from 671 to 494; also in controls from 568 to 317. However, results shows that those without cardiovascular disease at baseline, did not display a similar rate reduction over time. During this period, people with T1DM had a 1.34-fold (95% CI 1.24–1.46) higher adjusted hazard for incident AF than controls when adjusting for sociodemographic factors. This hazard was attenuated to 0.95 (95% CI 0.87–1.03) after also accounting for coronary, cerebrovascular, kidney disease and heart failure; among those with T1DM. In those, with several risk factors at baseline, we observed a hazard ratio from 1.61 (95%, 1.07–2.43), and there was also an indication of clear risk reduction in those with zero risk factors, albeit non-significant (HR 0.60, 95% CI 0.35–1.04). In the T1DM cohort, the first available value of hemoglobin A1c, systolic blood pressure, body mass index and estimated glomerular filtration rate were each independently associated with incident AF and we noticed a clear linear risk increase for several cardiometabolic risk factors.

**Conclusions:**

The crude incidence of AF was higher for persons with versus without T1DM, and declined significantly in both groups. Adjusting for data-derived predictors of AF attenuated higher risks, suggesting that the higher AF risk for persons with T1DM is driven by its common comorbidities.

**Supplementary Information:**

The online version contains supplementary material available at 10.1186/s12933-024-02561-z.

## Introduction

The associations between both type 1 and type 2 diabetes (T1DM; T2D) and cardiovascular conditions like atherosclerotic cardiovascular disease (ASCVD): coronary artery disease, cerebrovascular disease, peripheral arterial disease; and heart failure (HF) are well-documented [1–4]. While robust epidemiologic evidence demonstrates higher risk for atrial fibrillation/atrial flutter [5] with T2D [1, 3, 6], the associations with various risk factors with T1DMM has not been so extensively studied [7, 8]. In addition, the optimal levels of cardiometabolic risk factors and relative importance of both modifiable and non-modifiable risk factors, such as, age, hypertension, obesity, chronic ischemic heart disease, and heart failure, along with diabetes-specific factors such as elevated HbA1c, dyslipidemia, kidney disease, and cardiac autonomic neuropathy [9–14], may contribute to the increased risk in individuals with T1DM.

The present study, utilizing data from the Swedish National Diabetes Registry, builds upon previous analyses of atrial fibrillation (AF) associations with type 1 diabetes (T1DM) that were conducted with shorter follow-up durations [5, 7]. This analysis extends prior work by including a 50% longer follow-up period, spanning two decades marked by a rapid increase in the prevalence and severity of cardiometabolic risk factors associated with AF in individuals with T1DM [9]. The primary objectives were to assess standardized incidence rates of AF over this extended period, to evaluate the excess risk based on the number of risk factors within therapeutic target ranges, and to identify the relative prognostic importance of modifiable cardiometabolic risk factors. Additionally, the study aimed to investigate the impact of achieving optimal risk factor control on AF incidence, providing a more detailed understanding of their contributions to AF risk in individuals with T1DM.

## Methods

Access to the datasets in this study is available from the sources stated in the paper on request to the data providers, fulfilling the legal and regulatory requirements, and with approval from the Swedish Ethical Review Authority. The authors had full access to the complete data in the study and take responsibility for the integrity of the data and data analyses.

### Study design and support

This nationwide observational study was approved by the Ethical Review Authority (2020-04796). The participants with diabetes provided written informed consent for participation before entering the registry and the information for matched controls was retrieved from the government agency Statistics Sweden (SCB).

### Data sources and study cohort

**Fig. 1:**
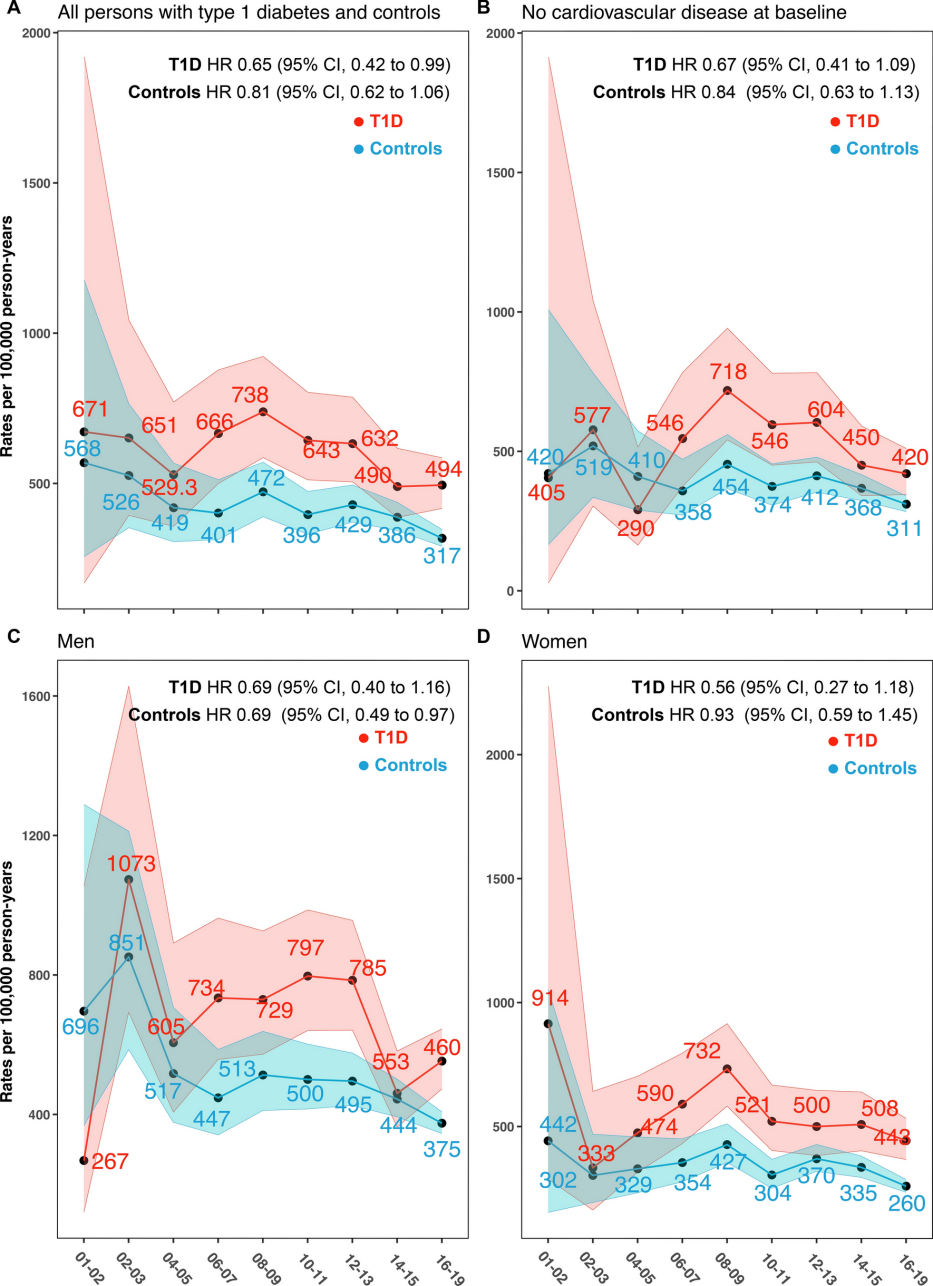
Standardized incidence rates for all outcomes among people with type 1 diabetes mellitus and matched controls. **A** and **B** shows age- and sex standardized incidence rates for all AF in people with type 1 diabetes mellitus compared with matched controls from the general population. The hazard ratio in the upper corner of each panel is constructed for T1DMM and controls separately, while adjusting for age, sex and time-period. Coefficients for the latter variable is raised by the power of 9 to yield a roughly 19-years of change in risk

Information on persons with T1DM is from the previously described Swedish National Diabetes Registry (NDR) [9–12]. Patients with T1DM were defined based on epidemiological criteria and the clinical assessment of physicians. The epidemiological definition of T1DM includes an age of onset before 30 years and exclusive treatment with insulin therapy. In approximately 5% of cases where data for the epidemiological definition were unavailable, the classification relied on the clinical assessment of physicians. This assessment incorporated factors such as age of onset, treatment modality, the presence of diabetes-associated autoantibodies, and C-peptide measurements to accurately differentiate between diabetes types. For each included person with T1DM with at least one entry in the registry between Jan 1, 2001 and Dec 31, 2019, five matched controls without diabetes were included. The controls were randomly selected from the Swedish general population provided by Statistics Sweden, and were matched for age, sex and county of residence. All study participants with AF at baseline, i.e., prior to inclusion in the registry were excluded from the present analyses. Participants with T1DMM who met the exclusion criterion were excluded from the analyses along with their corresponding matched controls. Controls were individually excluded, but without exclusion of their matched counterpart with T1DMM, if they met the specified exclusion criteria. A similar exclusion approach was performed to construct a cohort without AF and CVD at baseline for Fig. 1B.

### Outcomes

The outcome of incident AF was analyzed in persons with T1DM and in the matched controls. The outcomes were identified using data from the International Classification of Disease (ICD) versions 9 and 10. The specific ICD-codes are listed in supplementary Table 1.

### Statistical analyses

The study interval was divided into two-year epochs, with the exception of the last time-period that included three-years. The incidence rates were standardized using direct standardization to the age and distribution of the initial period. For the incidence rates, a ratio of the number of events occurring during each period (numerator) and the number of participants at risk during the same period (denominator), were calculated. Cox regression models to assess for change in risk over time were also performed a, using time-period as a covariate and a linear term. The coefficients for the linear terms (i.e., time-period) were exponentiated by the power of 9, along with the confidence intervals to calculate the roughly 19-year relative risk reduction separately for persons with T1DMM and for controls.

### Optimal risk factor levels

The association between specific cardiometabolic risk factors and AF was assessed using Cox proportional hazards models. These analyses were conducted solely on the cohort with T1DM, as data regarding cardiometabolic risk factors were only available for the cohort with T1DM and not for the control group. The following seven cardiometabolic risk factors were evaluated in these models; glycated hemoglobin (HbA1c, mmol/mol), systolic blood pressure (SBP, mmHg), diastolic blood pressure (DBP, mmHg), body mass index (BMI, kg/m^2^), estimated glomerular filtration rate (eGFR, ml/min/1.73m^2^), low-density lipoprotein cholesterol (LDL-C, mg/dL), and triglycerides (TG, mg/dL).

Univariable and multivariable Cox regression models were used to assess the relationship between each risk factor of interest and AF and were fitted with restricted cubic splines with three evenly spaced knots to detect non-linear relationships. The inclusion of extra knots for continuous predictors did not yield significant contributions to the regression models. Furthermore, the models were subjected to covariate adjustments to account for the following factors; age, sex, physical activity (0–5), ethnicity, marital status, income level, educational level, comorbidities, pharmacological treatment for cardiovascular conditions and smoking. By employing these models, we estimated non-linear risk associations for each individual risk factor and development of AF. An example of the Cox regression modeling approach specifically regarding the estimation of the optimal levels for example, for HbA1c, can be found in the Supplementary Material.

### Multifactorial risk factor assessment

We used Cox regression to assess the relationship between the co-presence of multiple risk factors beyond target range and the probability of developing AF. Individuals with T1DM were classified into different groups based on the number of baseline risk factors not within recommended evidence-based target levels. The risk factors were incorporated into the models as categorical variables, indicating whether the risk factors fell within the target ranges (yes/no). The risk factors included in these models, along with their respective cut-off values, include HbA1c ≥ 7.0%; SBP; ≥ 130 mmHg; DBP; ≥ 80 mmHg, current smoking, LDL-C ≥ 97 mg/dL, and the presence of normoalbuminuria, micro- or macroalbuminuria (henceforth denoted as albuminuria). To account for potential confounding factors, the Cox models were adjusted for sex, age, baseline comorbidities and socioeconomic variables. Furthermore, the models were modified to incorporate the duration of diabetes for persons with T1DM by assigning matched controls to a duration of zero years (i.e., dummy variable), while individuals with T1DM had their duration of T1DM centered around grand mean.

### Missing data

To address missing data, we used multiple imputation by chained equations (MICE). This approach allowed for the imputation of missing values based on observed data. The MICE method imputes missing values by prediction based on observed data. The imputation is only performed for individuals with T1DMM and cardiometabolic risk factors that are missing at baseline. The variables included in the imputation model can be found in Supplementary Table S2.

## Results

### Study population

The study population consisted of 36,069 individuals diagnosed with T1DM, alongside 165,705 matched controls. For participants with T1DM, the mean age was 34 years at the time of inclusion in the NDR. Roughly 3% of all patients with T1DM had coronary heart disease at baseline, compared to 0.4% for matched controls. Moreover, patients with T1DM had 1.1% had heart failure, 4.4% had end-stage kidney disease, 1.2% had acute myocardial infarction and 1.1% had peripheral arterial disease at baseline. Coexisting CV risk factors and conditions were approximately 2–6 times as frequent in people with T1DM compared to matched controls. See Table 1 for all baseline information for the study participants.

**Table 1:** Baseline characteristics for persons with type 1 diabetes, matched controls and diabetes according to age-categories

	Diabetes	Controls	Diabetes < 45	Diabetes > 75	Diabetes 45–54	Diabetes 55–64	Diabetes 65–74
Number of study participants	36,069	165,705	26,935	542	3067	4412	1113
Sex = women (%)	15,569 (43.2)	72,786 (43.9)	11,401 (42.3)	308 (56.8)	1339 (43.7)	1998 (45.3)	523 (47.0)
Age (mean (SD))	34 (16.48)	32 (15.05)	25 (7.75)	80 (4.81)	49 (2.87)	60 (2.43)	68 (2.77)
*Education (%)*							
Post-secondary education ≥ 12 years	6544 (18.1)	33,586 (20.3)	4712 (17.5)	68 (12.5)	898 (29.3)	631 (14.3)	235 (21.1)
Pre-secondary education ≤ 9 years	15,405 (42.7)	68,736 (41.5)	11,240 (41.7)	316 (58.3)	660 (21.5)	2723 (61.7)	466 (41.9)
Secondary education > 9 to 12 years	14,120 (39.1)	63,383 (38.3)	10,983 (40.8)	158 (29.2)	1509 (49.2)	1058 (24.0)	412 (37.0)
Civil = Married (%)	7406 (20.5)	30,740 (18.6)	3409 (12.7)	265 (48.9)	1576 (51.4)	1429 (32.4)	727 (65.3)
Ethnicity = Scandinavia (%)	32,984 (91.4)	149,183 (90.0)	24,914 (92.5)	495 (91.3)	2858 (93.2)	3662 (83.0)	1055 (94.8)
*Income family IQR (%)*							
IQR 1	8879 (24.6)	34,340 (20.7)	6823 (25.3)	293 (54.1)	771 (25.1)	617 (14.0)	375 (33.7)
IQR 2	7518 (20.8)	33,995 (20.5)	5427 (20.1)	184 (33.9)	808 (26.3)	705 (16.0)	394 (35.4)
IQR 3	9270 (25.7)	44,309 (26.7)	5612 (20.8)	46 (8.5)	826 (26.9)	2601 (59.0)	185 (16.6)
IQR4	10,402 (28.8)	53,061 (32.0)	9073 (33.7)	19 (3.5)	662 (21.6)	489 (11.1)	159 (14.3)
*Income IQR (%)*							
IQR 1	18,955 (52.6)	83,875 (50.6)	16,834 (62.5)	288 (53.1)	681 (22.2)	670 (15.2)	482 (43.3)
IQR 2	7068 (19.6)	28,885 (17.4)	4749 (17.6)	194 (35.8)	1024 (33.4)	750 (17.0)	351 (31.5)
IQR 3	6799 (18.8)	34,293 (20.7)	3275 (12.2)	40 (7.4)	797 (26.0)	2545 (57.7)	142 (12.8)
IQR 4	3247 (9.0)	18,652 (11.3)	2077 (7.7)	20 (3.7)	565 (18.4)	447 (10.1)	138 (12.4)
Coronary heart disease = Yes (%)	1043 (2.9)	730 (0.4)	76 (0.3)	158 (29.2)	207 (6.7)	359 (8.1)	243 (21.8)
Acute myocardial infarction = Yes (%)	435 (1.2)	305 (0.2)	32 (0.1)	85 (15.7)	86 (2.8)	134 (3.0)	98 (8.8)
Cerebrovascular disease = Yes (%)	346 (1.0)	403 (0.2)	56 (0.2)	58 (10.7)	65 (2.1)	98 (2.2)	69 (6.2)
Heart failure = Yes (%)	389 (1.1)	252 (0.2)	46 (0.2)	95 (17.5)	56 (1.8)	103 (2.3)	89 (8.0)
Hypertension = Yes (%)	2461 (6.8)	1506 (0.9)	780 (2.9)	216 (39.9)	494 (16.1)	603 (13.7)	368 (33.1)
Peripheral arterial disease = Yes (%)	381 (1.1)	111 (0.1)	20 (0.1)	63 (11.6)	76 (2.5)	127 (2.9)	95 (8.5)
Chronic obstructive pulmonary disease = Yes (%)	103 (0.3)	254 (0.2)	10 (0.0)	24 (4.4)	16 (0.5)	33 (0.7)	20 (1.8)
Dementia = Yes (%)	41 (0.1)	88 (0.1)	3 (0.0)	17 (3.1)	2 (0.1)	10 (0.2)	9 (0.8)
Alcoholism = Yes (%)	1204 (3.3)	3227 (1.9)	1016 (3.8)	7 (1.3)	82 (2.7)	81 (1.8)	18 (1.6)
End stage kidney disease = Yes (%)	1591 (4.4)	235 (0.1)	747 (2.8)	56 (10.3)	345 (11.2)	315 (7.1)	128 (11.5)
Age of onset of disease (y, mean (SD))	17.78 (10.65)		16.35 (9.88)	45.45 (23.36)	22.70 (12.23)	21.67 (13.27)	31.54 (18.32)
Duration of diabetes (y, mean (SD))	13.44 (13.31)		9.29 (8.37)	33.25 (20.74)	26.37 (12.42)	22.25 (16.94)	36.23 (17.70)
Glycated haemoglobin (Hba1c) (mmol/L; mean (SD))*	65.26 (18.20)		65.67 (19.10)	63.63 (16.83)	66.43 (15.51)	64.82 (17.72)	63.02 (14.71)
*HbA1c category (%)*							
48 mmol/mol	4729 (14.1)		4096 (15.2)	73 (13.5)	240 (7.8)	557 (12.6)	122 (11.0)
48 mmol/mol to 53 mmol/mol	4131 (12.3)		3283 (12.2)	78 (14.4)	331 (10.8)	596 (13.5)	175 (15.7)
54 mmol/mol to 58 mmol/mol	4224 (12.6)		3243 (12.0)	79 (14.6)	407 (13.3)	589 (13.3)	168 (15.1)
59 mmol/mol to 74 mmol/mol	12,040 (36.0)		9238 (34.3)	204 (37.6)	1336 (43.6)	1683 (38.1)	440 (39.5)
75 mmol/mol to 85 mmol/mol	4288 (12.8)		3422 (12.7)	61 (11.3)	452 (14.7)	509 (11.5)	134 (12.0)
86 mmol/mol	4045 (12.1)		3653 (13.6)	47 (8.7)	301 (9.8)	478 (10.8)	74 (6.6)
Smoker = Yes (%)	4533 (15.0)		4143 (15.4)	32 (5.9)	592 (19.3)	663 (15.0)	103 (9.3)
*Albuminuria (%)*							
No albuminuria	21,805 (87.5)		24,572 (91.2)	325 (60.0)	2270 (74.0)	3544 (80.3)	728 (65.4)
Normalized value	72 (0.3)		76 (0.3)	1 (0.2)	7 (0.2)	15 (0.3)	5 (0.4)
Microalbuminuria (3–30)	1934 (7.8)		1623 (6.0)	124 (22.9)	440 (14.3)	520 (11.8)	234 (21.0)
Macroalbuminuria (> 30)	1107 (4.4)		664 (2.5)	92 (17.0)	350 (11.4)	333 (7.5)	146 (13.1)
eGFR (ml/min/1.73m^2^, mean (SD))	102.45 (32.06)		123.27 (56.56)	61.32 (23.69)	84.78 (25.76)	106.89 (66.94)	69.82 (25.16)
Retinopathy = Yes (%)	5522		6600 (24.5)	344 (63.5)	1881 (61.3)	2151 (48.8)	768 (69.0)
Systolic blood pressure (mmHg, mean (SD))	123.58 (15.89)		120.17 (13.06)	142.38 (19.23)	133.91 (17.41)	130.24 (18.49)	142.47 (18.10)
Diastolic blood pressure (mmHg, mean (SD))	72.83 (9.16)		72.65 (9.05)	71.43 (10.70)	75.89 (9.31)	73.65 (9.32)	72.94 (9.68)
Total cholesterol (mmol/L mean (SD))	4.65 (1.03)		4.69 (1.08)	4.85 (1.18)	5.02 (1.08)	4.80 (1.11)	4.87 (1.14)
High-density lipoprotein colesterol (mmol/L mean (SD))	1.51 (0.45)		1.56 (0.48)	1.86 (0.67)	1.77 (0.58)	1.71 (0.58)	1.83 (0.63)
Triglycerides (mmol/L, mean (SD))	1.18 (0.97)		1.34 (1.25)	1.50 (1.60)	1.34 (1.14)	1.32 (1.36)	1.39 (1.72)
*Physical activity (%)*							
Never	1396 (8.1)		2574 (9.6)	140 (25.8)	460 (15.0)	628 (14.2)	223 (20.0)
< 1 time/week	2439 (14.1)		3749 (13.9)	112 (20.7)	522 (17.0)	679 (15.4)	196 (17.6)
1–2 time/week	3935 (22.7)		5796 (21.5)	99 (18.3)	651 (21.2)	859 (19.5)	239 (21.5)
3–5 time/week	5631 (32.5)		7652 (28.4)	80 (14.8)	674 (22.0)	1101 (25.0)	205 (18.4)
5 time/week	3927 (22.7)		7164 (26.6)	111 (20.5)	760 (24.8)	1145 (26.0)	250 (22.5)
Insulin method = manual insulin injection (%)	4753 (18.3)		4841 (18.1)	15 (2.9)	299 (9.8)	663 (15.1)	54 (5.0)
LDL-cholesterol (mmol/L, mean (SD))	2.63 (0.85)		2.59 (0.89)	2.44 (1.03)	2.71 (0.94)	2.56 (0.92)	2.49 (0.94)
S-creatinine (mg/dL, mean (SD))	75.83 (38.68)		67.97 (33.45)	102.73 (57.25)	89.26 (69.51)	80.41 (54.38)	95.91 (51.20)
Body mass index (kg/m^2^, mean (SD))	24.86 (6.97)		24.91 (4.15)	25.44 (4.34)	25.69 (4.23)	25.35 (4.26)	25.66 (4.34)

Values are mean (SD)

Income and eGFR is reported as median (inter quartile range [IQR])

Glomerular filtration rate was estimated using the Modification of Diet in Renal Disease Study Equation

Controls are individuals, matched for age, sex and county, who were randomly selected from the general population

^*^Concentrations of glycated hemoglobin are based on values from the International Federation of Clinical Chemistry

### Incidence rates and long-term trends

The median follow-up duration for persons with T1DM was 11.2 years (Table 1). A total of 821/36,069 (2.2%) incident AF events occurred in persons with T1DM and 2207/165,705 (1.3%) in matched controls. In persons with T1DM, the occurrence of AF demonstrated a decline in incidence rates from 671 to 494 cases per 100,000 person-years from the initial to final time period. These results are seen in the upper right corner of each panel in Fig. 1. Among the control group the incidence rate of AF decreased from 526 to 317 cases per 100,000 person-years (Fig. 1A). In individuals without cardiovascular disease at baseline, i.e., no coronary heart disease, cerebrovascular disease or heart failure, the results showed that persons with T1DM did not experience a similar decline in AF rate, 405–420 per 100,000 years, whereas matched controls experienced a decline from 420 to 311 cases per 100,000 person-years (Fig. 1B). The declines in AF between men and women were similar, 1073 (2nd time-period) to 460 and 914–442 per 100,000 person years, respectively (Fig. 1C, D). Hazard ratios in the upper corner of each figure panel shows the change in risk over time for a roughly 20-year time-period. Risk for AF has decreased by 35% in persons with T1DMM over the entire follow-up period.

### Excess risk for atrial fibrillation

Table 2 displays sociodemographic predictors and comorbidities of risk for AF with hazard ratios, confidence intervals and significance level for a Cox model that includes all of the variables displayed in Table 2. These models did not include covariates of non-cardiovascular comorbidities nor pharmacological treatment. The Cox model with sociodemographic covariates displayed an excess risk for T1DM (aHR 1.34, 95% CI 1.24–1.46), when adjusted for both sociodemographic and comorbidities, T1DM was not associated with AF risk (aHR 0.95; 95% CI 0.87–1.03). The Cox regression model that included the entire cohort along with both sociodemographic covariates and comorbidities showed that coronary heart disease was associated with 1.49 aHR (95% CI 1.29–1.72), heart failure 1.86 aHR (95% CI 1.47–2.35) and end-stage kidney disease displayed a risk increase of 1.77 aHR (95% CI 1.46–2.14).

**Table 2:** Cox models with varying covariate adjustment and time periods

Characteristic	Entire cohort			Date < 2010-01-01			Date > 2010-01-01		
	HR^1^	95% CI^1^	p-value	HR^1^	95% CI^1^	p-value	HR^1^	95% CI^1^	p-value
Age	1.10	1.09, 1.10	< 0.001	1.10	1.10, 1.10	< 0.001	1.09	1.08, 1.10	< 0.001
*Group*									
Controls	–	–		–	–		–	–	
T1D	1.34	1.24, 1.46	< 0.001	1.35	1.24, 1.48	< 0.001	1.25	0.97, 1.61	0.084
Sex	0.52	0.48, 0.56	< 0.001	0.52	0.48, 0.56	< 0.001	0.56	0.44, 0.71	< 0.001
*Education*									
Pre-secondary education < 9 years	–	–		–	–		–	–	
Post-secondary education > 12 years	1.21	1.09, 1.34	< 0.001	1.18	1.05, 1.32	0.004	1.51	1.10, 2.08	0.012
Secondary education > 9 to 12 years	1.30	1.19, 1.41	< 0.001	1.28	1.16, 1.40	< 0.001	1.53	1.17, 2.01	0.002
*Ethnicity*									
All other countries	–	–		–	–		–	–	
Scandinavia	1.68	1.42, 1.97	< 0.001	1.70	1.42, 2.03	< 0.001	1.47	1.01, 2.15	0.047
*Income quartiles*									
IQR1	–	–		–	–		–	–	
IQR2	0.86	0.78, 0.94	0.001	0.87	0.79, 0.96	0.005	0.87	0.63, 1.19	0.4
IQR3	0.55	0.50, 0.61	< 0.001	0.57	0.52, 0.64	< 0.001	0.44	0.31, 0.61	< 0.001
IQR4	0.80	0.71, 0.91	< 0.001	0.87	0.76, 0.99	0.036	0.58	0.42, 0.81	0.001
*Marital status*									
All other marital statuses	–	–		–	–		–	–	
Married	1.12	1.04, 1.21	0.002	1.11	1.02, 1.20	0.013	1.24	0.97, 1.58	0.091

Characteristic	Entire cohort (all covariates)			Date < 2010-01-01			Date > 2010-01-01		
	HR^1^	95% CI^1^	p-value	HR^1^	95% CI^1^	p-value	*HR*^1^	95% CI^1^	p-value
Age	1.09	1.09, 1.09	< 0.001	1.09	1.09, 1.09	< 0.001	1.08	1.08, 1.09	< 0.001
*Group*									
Controls	–	–		–	–		–	–	
T1D	0.95	0.87, 1.03	0.2	0.96	0.88, 1.05	0.4	0.88	0.68, 1.15	0.4
Sex	0.51	0.47, 0.55	< 0.001	0.50	0.46, 0.55	< 0.001	0.53	0.42, 0.67	< 0.001
*Education*									
Pre-secondary education < 9 years	–	–		–	–		–	–	
Post-secondary education > 12 years	1.03	0.93, 1.15	0.6	0.99	0.89, 1.11	0.9	1.44	1.05, 1.98	0.025
Secondary education > 9 to 12 years	1.11	1.02, 1.21	0.020	1.08	0.98, 1.18	0.11	1.45	1.10, 1.90	0.007
*Ethnicity*									
All other countries	–	–		–	–		–	–	
Scandinavia	1.38	1.17, 1.62	< 0.001	1.38	1.15, 1.65	< 0.001	1.25	0.86, 1.83	0.2
*Income quartiles*									
IQR1	–	–		–	–		–	–	
IQR2	0.88	0.80, 0.96	0.005	0.89	0.81, 0.98	0.017	0.88	0.64, 1.21	0.4
IQR3	0.73	0.66, 0.81	< 0.001	0.77	0.69, 0.86	< 0.001	0.54	0.39, 0.76	< 0.001
IQR4	0.88	0.78, 0.99	0.031	0.94	0.83, 1.07	0.4	0.63	0.46, 0.88	0.006
*Marital status*									
All other marital statuses	–	–		–	–		–	–	
Married	1.00	0.93, 1.08	> 0.9	0.97	0.90, 1.05	0.5	1.19	0.93, 1.51	0.2
Coronary heart disease	1.49	1.29, 1.72	< 0.001	1.51	1.30, 1.76	< 0.001	1.30	0.85, 1.97	0.2
Heart failure	1.86	1.47, 2.35	< 0.001	1.75	1.36, 2.27	< 0.001	2.70	1.46, 5.02	0.002
Cerebrovascular disease	1.19	0.94, 1.50	0.2	1.10	0.85, 1.44	0.5	1.83	1.07, 3.13	0.027
End stage renal disease	1.77	1.46, 2.14	< 0.001	1.79	1.46, 2.19	< 0.001	1.53	0.85, 2.78	0.2

*HR* hazard ratio, *CI* confidence interval

### Associations between risk factor levels and risk of atrial fibrillation among persons with T1DM

**Fig. 2:**
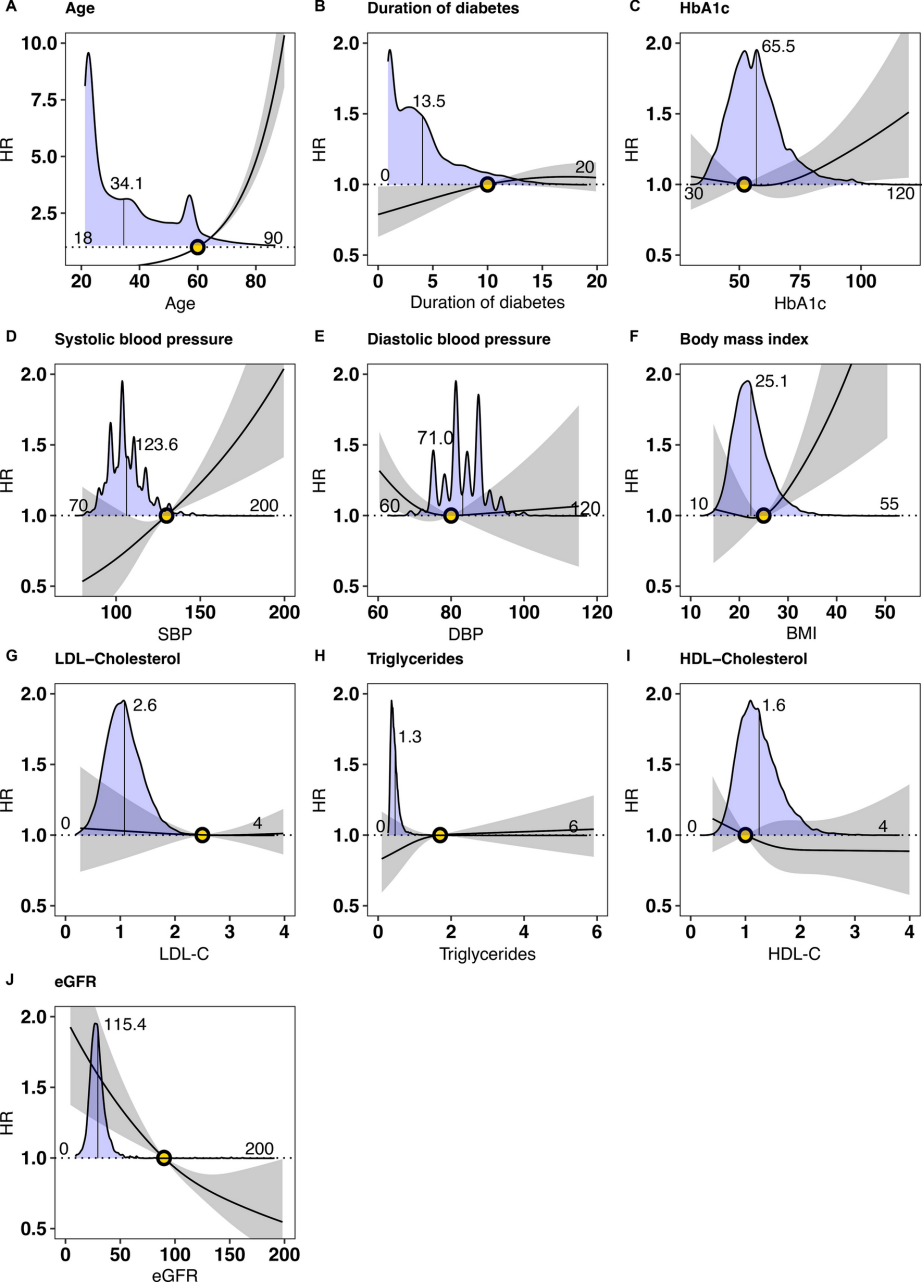
Association between risk factor levels and AF in persons with type 1 diabetes mellitus. To capture potential non-linear associations, the Cox regression models incorporated restricted cubic splines with three evenly spaced knots The dark lines indicate the hazard function and the shaded areas 95% confidence intervals. The following cut-off levels were used for risk factors: age > 60 years, duration of diabetes > 10 years, glycated hemoglobin (≥ 7.0% (52 mmol/mol)), SBP (≥ 130 mmHg), DBP (≥ 80 mmHg), and eGFR (≤ 90 ml/min/1.73 m^2^), BMI ≥ 27.5 kg/m.^2^, LDL-C (≥ 96 mg/dL), HDL-C (≤ 60 mg/dL), and triglycerides (≥ 151 mg/dL)

Figure 2 presents the associations between various factors and the risk association of incident AF in individuals with T1DM. This figure includes a depiction of the diabetes guideline target level using circles. In addition, the figure includes density plots for the data used in the Cox models, these density plots also include imputed values. As depicted in Fig. 2C, an association was observed between higher levels of HbA1c and higher risk of AF, albeit the risk association was significant first at higher levels. Moreover, the hazard risk reveals that lower levels of HbA1c also tended to be associated with a higher risk of AF, although not significant. Age demonstrated a substantial risk association for AF in T1DMM.

Figure 2D and J depict near-linear associations between higher levels of SBP and lower levels of eGFR, respectively and the risk of AF. Similarly, Fig. 2F shows that higher levels of BMI are significantly associated with higher risk of AF. Lipids such as LDL–C, HDL–C and TG were not associated with AF risk (Fig. 2G–I).

### Multifactorial risk factors within target levels and relative importance to prognostic modeling of individual risk factors of AF in T1DM

**Fig. 3:**
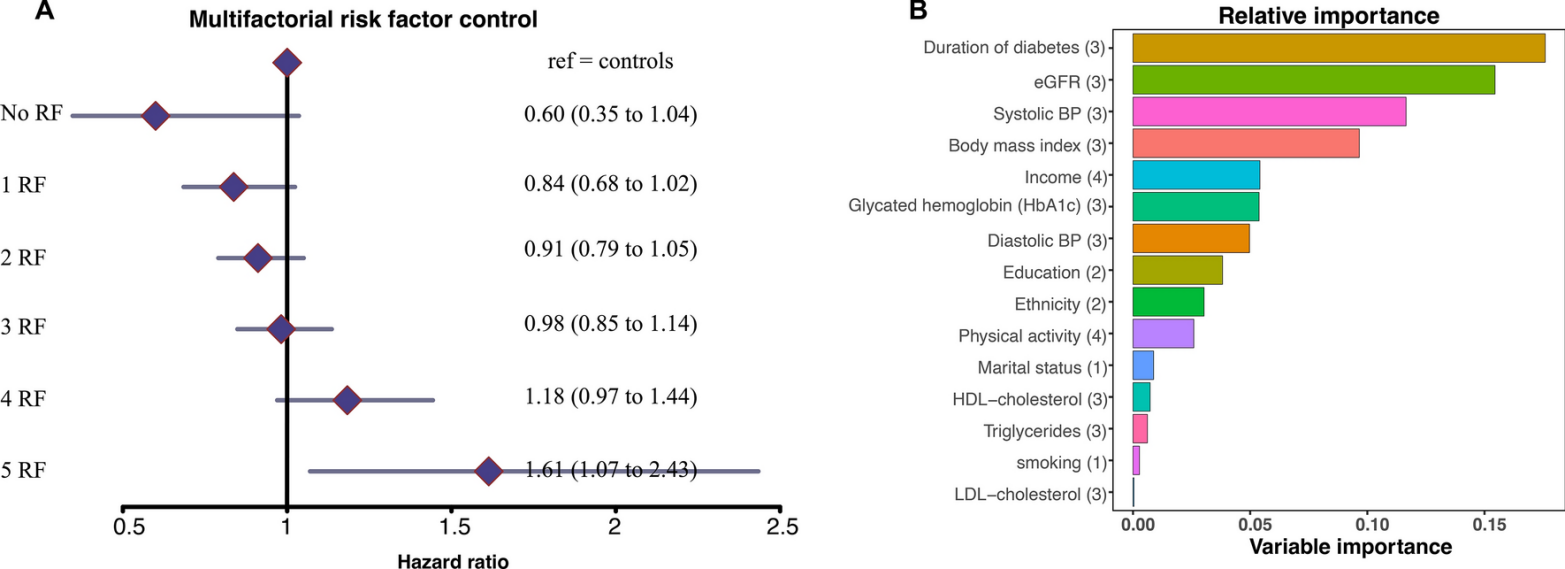
Adjusted hazard ratios for AF, according to number of cardiovascular risk factors beyond target in people with type 1 diabetes mellitus and relative importance of variables from Cox proportional hazards model. **A** People with type 1 diabetes mellitus are stratified into six different subgroups based on the number of risk factors, ranging from 0 to 5, beyond target. **B** Variables with a high importance estimate have a higher partial graded effect predictive to the model and are deemed important

Figure 3A presents the adjusted hazard ratios for AF, comparing persons with T1DM categorized into 5 groups based on the numbers of risk factors within target levels at baseline with their matched controls (for whom risk factor level data are not available). The following five risk factors were considered, with respective target-level cut-off values: HbA1c > 7.0%; SBP > 130 mmHg; DBP > 80 mmHg, albuminuria, non-smoker; and LDL-C > 97 mg/dl.

Consistently, for each additional risk factor not within the target range, there was incremental risk of AF. Individuals with T1DM who had all 5 risk factors outside the target range at baseline had an adjusted hazard ratio of 1.61 (95% CI 1.107–2.43) for incident AF when compared with their controls. Conversely, those who had all risk factors within the target range exhibited a HR of 0.60 (95% CI 0.35–1.04) compared with their controls, and persons with only one risk factor outside the target range at baseline had a HR of 0.84 (95% CI 0.68–1.02) for incident AF.

Figure 3B showed that the duration of diabetes is the most important relative risk factor for AF and explains roughly 20% of the entire prediction, it was almost as significant as the second most important relative risk factor, eGFR that explains roughly 15%. Followed closely by systolic blood pressure and BMI that together roughly explains 20% of the prediction model. The least important relative risk factors were marital status, smoking and lipids such as TG, HDL–C and LDL–C. This model includes time-updated age as the timescale in the Cox model.

### Trends in cardiovascular risk factors and cardiorenal disease

**Fig. 4:**
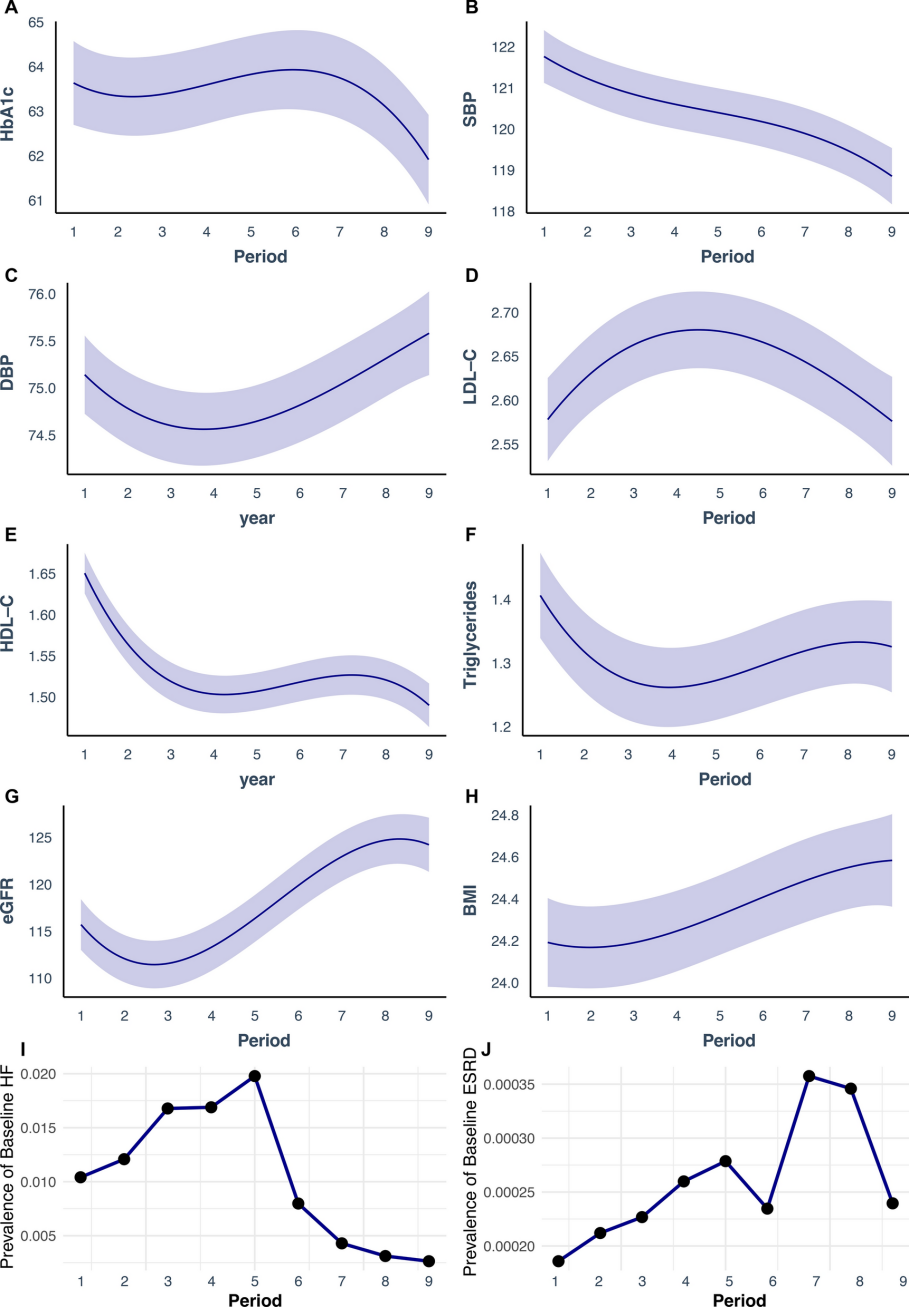
Linear regression for changes in baseline characteristics of cardiometabolic risk factors, while adjusting for covariates, and changes in prevalence of baseline heart failure and end-stage renal disease. Legend: Multivariable linear regression was used to assess changes in cardiometabolic risk factors for the type 1 diabetes population at baseline, according to time period. Prevalence was measured for heart failure and end-stage kidney disease, at baseline, according to time period

Figure 4A–J shows the change of cardiovascular risk factors and the prevalence of HF and ESRD, for each time period. HbA1c, SBP, eGFR and LDL–C has improved over time, whereas, BMI, DBP and HDL–C has worsened. In addition, the prevalence of HF at baseline has improved with a sharp decline from the fifth time period and onwards, while prevalence of ESRD has increased steadily in the T1DM population.

## Discussion

Results from this Swedish nationwide study over the past two decades demonstrates that AF incidence has declined significantly in persons with T1DMM (26.4% reduction) and in the cohort of matched controls (55% reduction). The relative risk reduction demonstrates a 35% decrease in risk for individuals with T1DMM, compared to a 19% reduction observed in the control group. Subgroup analyses revealed that individuals with T1DMM that had no CVD at baseline, did not display a significantly reduction over time, indicating that AF in T1DMM, is primarily driven by CVD, The excess risk of AF in individuals with T1DM, observed in age- and sex-adjusted analyses, was attenuated after multivariable adjustment. This attenuation is presumably explained by the high prevalence of cardiometabolic comorbidities, particularly ESRD, hypertension, and obesity, which contribute to atrial remodeling and promote conditions favorable for AF development. These risk factors mechanisms highlight the interplay between T1DM and its associated comorbidities in driving AF risk. After adjusting for predictors of AF, including age, sex, body mass index, blood pressure, HbA1c, estimated glomerular filtration rate, and cardiovascular comorbidities at baseline, T1DM was no longer associated with an increased risk of AF. Thus, the higher AF risk in persons with T1DM is largely attributable to common comorbidities rather than T1DM itself.

In the context of previously published research, our findings reveal that after thorough adjustment for concomitant risk factors, the observed relationship between T1DM and AF diminishes. This underscores that the elevated AF risk in individuals with T1DM is not due to T1DM itself but rather the collective impact of comorbidities such as ESRD, hypertension, and obesity. ESRD contributes to AF through systemic inflammation, oxidative stress, and volume overload, all of which promote atrial fibrosis, structural remodeling, and electrical conduction abnormalities. Furthermore, uremic toxins in ESRD patients can directly impair myocardial electrophysiology, exacerbating arrhythmogenic risk. Hypertension, another common comorbidity, accelerates left ventricular hypertrophy and diastolic dysfunction, indirectly increasing left atrial pressure and size, both of which are known predictors of AF. The high prevalence of ESRD in this population underscores its importance, indicating a pressing need for targeted interventions to mitigate this risk. Hyperglycemia in T1DM exacerbates oxidative stress and advanced glycation end-product (AGE) accumulation, leading to endothelial dysfunction and inflammation, which can amplify atrial fibrosis and electrical remodeling. Chronic hyperglycemia and insulin resistance may also impair autonomic balance, increasing susceptibility to AF. Furthermore, impaired renal function, as seen in ESRD, alters electrolyte homeostasis, particularly potassium and magnesium levels, which are critical for maintaining atrial electrical stability. These interconnected mechanisms point to the pressing need for comprehensive management of cardiometabolic and renal health in individuals with T1DM to mitigate the heightened AF risk.

The non-significant excess risk can be due to improved management and treatment of cardiometabolic risk factors [13–15] such as HbA1c, blood pressure, lipid levels, and reduced smoking within the population. Improved management of cardiometabolic risk factors presumably decreases the risk of atrial remodeling and AF, while risk factor improvement also decreases the risk of diabetes-related complications, which in turn reduces incidence rates of AF.

The results demonstrate that for each risk factor within range, there is a stepwise lower risk for AF for people with T1DM. Persons with T1DM and 0–1 risk factors out of range did not have higher risk for AF compared with their matched controls. Given that the “in range” risk factor status for the participants with T1DM may be either de novo or be in the context of medical treatment to target, the conclusion should not be that treating to these targets will reduce AF risk, but rather raise the hypothesis that treating to these targets for those with levels beyond ideal may improve AF risk.

### Limitations

Information on cardiometabolic data and other CV risk factors prevalence and proportion in range for the control participants was not available, which made it impossible to adjust for the risk factors in the regression models comparing those with and without T1DM. Atrial fibrillation and flutter was coded as one outcome due to possibility of overlap and inaccuracy between the two conditions in the patient registry. We used imputed baseline values of cardiometabolic risk factors for persons with T1DM in the regression models, which may be considered as a limitation. The T1DM population in the NDR has quite high coverage for risk factors, thus, a relatively few number of values are imputed with MICE, primarily lipids. The results are contingent on the chosen model and may differ when applying alternative statistical methods. The study did not differentiate between individuals whose risk factors were naturally within desired ranges, without any deliberate intervention, and those who underwent medical treatments to achieve the desired levels. As a result, this study does not evaluate the management of risk factors but rather focuses on their proportion within acceptable ranges and that association with AF risk. Additionally, no adjustments were applied for conducting multiple tests.

## Conclusions

Over the 20-year study period, the crude incidence of AF was higher for persons with versus without T1DM, and declined significantly in both groups. Adjusting for data-derived predictors of AF, including age, sex, body mass index, blood pressure, hemoglobin A1c, estimated glomerular filtration rate and heart failure, T1DM was no longer associated with AF risk, suggesting that the higher AF risk for persons with T1DM is driven by its impact on common comorbidities.

## Supplementary Information

Additional file1 (PDF 114 kb)

## Data Availability

No datasets were generated or analysed during the current study.

## Abbreviations

BMI: Body mass index
HbA1c: Glycated hemoglobin
SBP: Systolic blood pressure
DBP: Diastolic blood pressure
LDL-C: Low-density lipoprotein cholesterol
HDL-C: High-density lipoprotein cholesterol
TG: Triglycerides
eGFR: Estimated glomerular filtration rate
ICD: The International Classification of Disease
AF: Atrial fibrillation and atrial flutter
T1DM: Type 1 diabetes mellitus
ESRD: End-stage renal disease
HF: Heart failure
