# Supplementary material for: Cardiometabolic risk factors and disease trends for atrial fibrillation in individuals with type 1 diabetes: a nationwide registry study
Source: Cardiovasc Diabetol. 2025 Mar 12;24:117. doi: 10.1186/s12933-024-02561-z (PMC11905555; doi:10.1186/s12933-024-02561-z)
Supplement: Supplementary file 1 — Additional file1 (PDF 114 kb) [file 12933_2024_2561_MOESM1_ESM.pdf]

Cardiometabolic risk factors and disease trends for atrial fibrillation in individuals with type 1 diabetes: a nationwide registry study

Supplemental material

**Table S1. ICD-10 Codes Used to Define Baseline Conditions and Outcomes**		
**Outcomes**	**ICD-9**	**ICD-10**
**Cardiac arrhythmias**		
Atrial fibrillation and atrial flutter	I427D, I4273	I480, I481, I482, I483, I484, I489
**Comorbidities**		
Coronary heart disease	410-414	I20-I25
Stroke	431-434, 436	I61-I64
Heart failure & cardiomyopathies	425, 421, 428	I50, I42
Hypertension	401, 402, 403, 404, 405	I109, I11, I120, I129, I132, I139, I130, I131, I159, I150, I152, I158
Peripheral arterial disease	440C, 443X	I702, I739
Dementia	294B	F00, F01, F02, F039
End-stage renal disease	5818. 584, 585, 583, 590, 586, 593X, 791X, V42A, 250D, 403, V45B, V56	N179, N178, N170, N172, N189, N181, N182, N183, N184, N185, N199, Z940, DR016, N083, E112C, E112C, E112W, E112X, E102, E102X, E132, E242, E122, E112A, E112B, I120, Z992, DR013, DR023, DR056, Z941, Z492
Cancer		C00–C97
COPD	496, 491	J44
Alcoholism	291, 292, 303, 305A	F10
* Includes ICD-codes as main diagnosis and up to 6 contributory causes for outcomes and comorbidities		

**Table S2 Variables Used in the Imputation Algorithm.**
**Variables:** Age, sex, age at onset of diabetes, clinicians diagnosis type, epidemiological definition of diabetes, treatment of diabetes, method of insulin treatment, systolic blood pressure, diastolic blood pressure, body weight, body length, glycated hemoglobin (HbA1c), total cholesterol, triglycerides, HDL-cholesterol, LDL-cholesterol, albuminuria, s-creatinine, retinopathy, smoking status, physical activity, county, body mass index, marital status, education, ethnicity, income, family income, eGFR, history of acute myocardial infarction, coronary heart disease, heart failure, hypertension, peripheral arterial disease, chronic obstructive pulmonary disease, dementia, alcoholism, end-stage renal disease, cancer and stroke
